# Echocardiographic partition values and prevalence of left ventricular hypertrophy in hypertensive Nigerians

**DOI:** 10.1186/1471-2342-6-10

**Published:** 2006-08-29

**Authors:** Adewole A Adebiyi, Okechukwu S Ogah, Akinyemi Aje, Dike B Ojji, Adedeji K Adebayo, Olulola O Oladapo, Ayodele O Falase

**Affiliations:** 1 Division of Cardiovascular Medicine, Department of Medicine, University College Hospital, Ibadan, PMB 5116 Ibadan, Oyo State, Nigeria/Division of Cardiovascular Medicine, Department of Medicine, College of Medicine, University of Ibadan, Nigeria; 2 Division of Cardiovascular Medicine, Department of Medicine, University College Hospital, Ibadan, PMB 5116 Ibadan, Oyo State, Nigeria

## Abstract

**Background:**

Left ventricular hypertrophy (LVH) is a well known independent risk factor for cardiovascular events. It has been shown that combination of left ventricular mass (LVM) and relative wall thickness (RWT) can be used to identify different forms of left ventricular (LV) geometry. Prospective studies have shown that LV geometric patterns have prognostic implications, with the worst prognosis associated with concentric hypertrophy. The methods for the normalization or indexation of LVM have also recently been shown to confer some prognostic value especially in obese population. We sought to determine the prevalence of echocardiographic lLVH using eight different and published cut-off or threshold values in hypertensive subjects seen in a developing country's tertiary centre.

**Methods:**

Echocardiography was performed in four hundred and eighty consecutive hypertensive subjects attending the cardiology clinic of the University college Hospital Ibadan, Nigeria over a two-year period.

**Results:**

Complete data was obtained in 457 (95.2%) of the 480 subjects (48.6% women). The prevalence of LVH ranged between 30.9–56.0%. The highest prevalence was when LVM was indexed to the power of 2.7 with a partition value of 49.2 g/ht^2.7 ^in men and 46.7 g/ht^2.7 ^in women. The lowest prevalence was observed when LVM was indexed to body surface area (BSA) and a partition value of 125 g/m^2 ^was used for both sexes. Abnormal LV geometry was present in 61.1%–74.0% of our subjects and commoner in women.

**Conclusion:**

The prevalence of LVH hypertensive patients is strongly dependent on the cut-off value used to define it. Large-scale prospective study will be needed to determine the prognostic implications of the different LV geometry in native Africans.

## Background

Left ventricular hypertrophy (LVH) either diagnosed by electrocardiography or echocardiography is now well known as an independent risk factor for cardiovascular events. Echocardiographic LVH is diagnosed based on cut-off values developed from population based studies in which LV mass is indexed to body surface area (BSA), height or height raised to the power of 2.7, the allometric growth rate of the heart. Based on recent work by Koren et al [1] and Ganau et al [2], combination of LVM and relative wall thickness (RWT) can now be used to identify different forms of LV geometry. Prospective studies have shown that LV geometric patterns have prognostic implications, with the worst prognosis associated with concentric hypertrophy [3]. The methods for the normalization or indexation of LV mass have also recently been shown to confer some prognostic value especially in obese population [4,5].

Many studies have used different partition values for left ventricular mass (LVM) in the classification of LV geometry. The purpose of this study is therefore to assess the influence of various published partition values for LVM in the diagnosis of LVH and LV geometry in native Africans.

## Methods

The study was carried out at the Cardiology clinic of the University College Hospital, Ibadan, Nigeria. It was an observational cross-sectional study, which was conducted within a two-year period. Hypertensive patients were eligible for the study if they fulfilled the following criteria: no evidence valvular abnormality (aortic or mitral value disease) or congestive heart failure. Subjects with sickle cell disease, diabetes mellitus, renal failure, and ischaemic heart disease were also excluded from the study. Both treated and untreated hypertensive subjects were recruited.

All the subjects gave informed consent before they were enrolled into the study. Ethical clearance was obtained from the joint University of Ibadan and University College Hospital Ibadan ethical committee.

### Clinical evaluation

Baseline clinical and demographic characteristics were obtained from the subjects. These included date of birth, age, gender, history of diabetes, and history of smoking and alcohol use. Blood pressure measurements were obtained according to standard guidelines with a mercury sphygmomanometer (Accosson London). Systolic and diastolic blood pressures were measured at Korotkoff sounds phases I and V respectively. Blood pressure was measured at the right arm three (3) times and averaged after a 5 minutes rest. Blood pressure 140/90 and above was taken as hypertension[6]. Subjects were weighed without shoes and in light clothing on a standard beam balance. Height was measured to the nearest centimetre using anthropometrical plane with subjects not putting on shoes or headgear. Body mass index (BMI) was calculated using the formula: BMI = Weight (kg)/(height). Body surface area (BSA) was calculated using the formula of Dubois.[7]

### Echocardiography

M-mode, 2D and Doppler echocardiography were performed using a standard protocol and an ALOKA SSD echocardiography machine (Aloka Co. Ltd., Tokyo, Japan). Two dimensional guided M- mode measurements were made according to the recommendations of the American Society of Echocardiography (ASE)[8]. LV internal dimension, posterior wall thickness and interventricular septal thickness were measured at end-diastole and end-systole. Where optimal M-mode imaging could not be obtained, 2D linear measurements were obtained according to the ASE criteria [8]. Left atrial end systolic diameter was obtained from the trailing edge of the posterior aortic – anterior left atrial complex. Measurements were obtained in up to 3 cardiac cycles according to the ASE convention [8]. Two experienced physicians performed the echocardiography. In our laboratory, the intra-observer concordance correlation coefficient ranged from 0.76 to 0.98 while that of the inter-observer concordance ranged from 0.82 to 0.96[9]

### Calculation of derived variables

Left ventricular mass was calculated using the formula of Devereux and Reichek.[10] This has been shown to yield LVM closely related to autopsy measurements (r = 0.90)[11] and has good interobserver reproducibility (ρ = 0.93) in one study[12]. Relative wall thickness (RWT) was derived from 2 X posterior wall thickness/LV internal diameter. Increased RWT was considered to be present when RWT exceeded 0.43. This represents the 97.5^th ^percentile in normal subjects [13].

We assessed left ventricular hypertrophy using various published partition values.

Partition values for LVM normalized for BSA were:

□ 125 g/m^2 ^for both men and women[1],

□ 116 g/m^2 ^for men and 104 g/m^2 ^for women[14],

□ 125/m^2 ^for men and 110 g/m^2 ^for women[15],

□ 131 g/m^2 ^for men and 100 g/m^2 ^for women[16].

Partition values for LVM indexed for height were:

□ 126 g/m for men and 105 g/m for women[17]

□ 143 g/m for men and 102 g/m for women[16],

Values for LVM raised to the allometric growth rate of 2.7 were:

□ 51 g/m^2.7 ^for both men and women[17]

□ 49.2 g/m^2.7 ^for men and 46.7 g/m^2.7 ^for women[17]

Left ventricular geometric was defined as follows: Normal geometry, when LVMI and RWT were normal; Concentric remodeling, when LVMI was normal and RWT increased; Eccentric hypertrophy, when LVMI was increased but normal RWT; and Concentric hypertrophy, when both LVMI and RWT were increased [2].

### Statistical analysis

SPSS version 11.0 software (SPSS, Chicago, IL, USA) was used in the analysis of the data. Continuous variables were expressed as mean ± SD while categorical variables were expressed as counts (percentages). Normality of continuous variables was assessed using the Kolmogorov- Smirnov statistics. Comparison between two groups was assessed by the Students t- test for independent variables while the χ^2 ^analysis was used to compare proportions. A 2-tailed p-value of 0.05 was assumed statistically significant.

## Results

### Clinical characteristics

The clinical characteristic of our study subjects is as shown in Table 1. A total of four hundred and eighty hypertensive subjects who met the inclusion criteria were recruited for the study. Twenty three (23) subjects were dropped from the final analysis because of incomplete data or inadequate echocardiogram. Four hundred and fifty seven subjects were included in the analysis (95.2%). These were two hundred and thirty five (235) men and two hundred and twenty two (222) women constituting 51.4% and 48.6% respectively. The mean ages for men and women were similar (56.0 ± 13.0 vs 55.5 ± 13.5, p = 0.672). The men were taller and had a greater body surface area than the women (P < 0.0001). Clinic blood pressures were similar in both sexes.

### Echocardiographic measurements

Table 2 shows the echocardiographic measurements in the subjects. The echocardiographic LV parameters were generally higher in men than in women except for the indexes of LV systolic function.

Table 3 depicts the prevalence of left ventricular hypertrophy and abnormal LV geometry in the hypertensive subjects. The prevalence of left ventricular hypertrophy based on LVM above the threshold value ranged between 30.9% and 56%. The lowest prevalence is when LVM was indexed to BSA and a partition value of 125 g/m^2 ^was used for both sexes. Highest prevalence occurred when LVM was indexed to height raised to the power of 2.7 and partition value of 49.2 g/ht^2.7 ^in men and 46.7 g/ht ^2.7 ^in women. The prevalence of abnormal LV geometry ranged from 61.1% to 74%.

Table 4 shows the distribution of LV Geometry according to gender. Abnormal LV geometry is significantly higher in women than men except when LVM was indexed to height^2.7 ^with a partition value of 51 g/height^2.7 ^for both sexes.

## Discussion

It is now well established that LVH either determined by electrocardiography or echocardiography is a strong predictor of poor prognosis in cardiovascular disorders independent of traditional risk factors. Various authors have used different partition values to define increased LVM. This includes BSA [18], BSA raised to the power of 1.5; height, height raised to the power of 2.0, 2.13, 2.7[17] and 3.0. Furthermore, combination of indexed LVM and RWT has been used to define geometry.

This study is the first to look at the impact of various cut-off values for LVM on the prevalence of LVH and abnormal LV geometry in hypertensive native Africans.

Our study shows that the prevalence of LVH ranges between 30.9–56.0%. We also observed that the highest prevalence was when LVM was indexed to the power of 2.7 with a partition value of 49.2 g/ht^2.7 ^in men and 46.7 g/ht^2.7 ^in women. The lowest prevalence was observed when LVM was indexed to BSA and a partition value of 125 g/m^2 ^was used for both sexes. We also report that abnormal LV geometry is present in 61.1%–74.0% of our subjects. Eccentric LV geometry was the commonest abnormal geometry in our hypertensive population (17.5–30.4%) while concentric LV geometry was present in 3.3–25.6% of the subjects. Normal geometry was seen in 26–38.9%. Abnormal geometry was more common in women in all the partition values.

Our finding is similar to those of other workers. Wachtell and his colleagues [19] in the LIFE multi-centre study group studied 941 stage I-III hypertensive subjects. They reported a 42–78% prevalence of LVH and 63–86% prevalence of abnormal LV geometry. Fifteen to forty percent (15–40%) of their subjects had normal geometry. Eccentric LV geometry was also the commonest abnormal LV geometry in their study.

In a cross-sectional study, Coca et al studied 946 hypertensive subjects recruited randomly from thirty nine (39) primary health care centres in Spain. They reported a 59.2–72.7% prevalence of LVH depending on the criteria used. They also noted that the prevalence of LVH was higher in men using the Framingham criteria but higher in women using de Simone et al criteria. Eccentric LVH was the commonest abnormal criteria in their study (51.3–54.1%) independent of the criteria used. Normal geometry was seen in 20.8–29.7% of their subjects. Overall, 70.3–79.2% of their hypertensive subjects had abnormal geometry.

Cuspidi and his co-workers[20], using six different echocardiographic criteria, studied 611 consecutive hypertensive subjects in Italy. The prevalence of LVH in their study was 18.6–42.7%. Eccentric LV geometry was commonest and abnormal LV geometry was more common in women than the men. They also observed that LVM correlated positively well with BSA, height, and height^2.7 ^and carotid intima-media thickness.

Three groups studied the influence of different partition values on the prevalence of LVH in newly diagnosed untreated hypertensive subjects.

In the study of 165 untreated essential hypertensive subjects by Ganau et al[2], 52% of their subjects had normal geometry, 13% had concentric remodeling, 27% had eccentric hypertrophy while only 8% had the "typical" hypertensive left ventricular hypertrophy. Furthermore, they documented that systemic hemodynamics paralleled ventricular geometry, with the highest peripheral resistance in the groups with concentric remodeling and hypertrophy, whereas cardiac index was super-normal in those with eccentric hypertrophy and low normal in patients with concentric remodeling[2].

Gosse et al[21] studied 363 untreated patients using three partition values. They reported the prevalence of LVH as 48.2–50.4%. The authors concluded that a cut-off value of 53 g/m^2.7 ^in men and 47 g/m^2.7 ^in women corresponded to a cardiovascular risk indicated by daytime systolic BP >= 135 mmHg. Another study reported a prevalence of 9–25% in newly diagnosed hypertensive subjects [22].

Similar to the observation of Wachtell et al, most of the criteria yielded unequal distribution of abnormal LV geometry between men and women in our study. But unlike their finding (where four criteria gave similar distribution in men and women), only one criterion gave similar distribution in men and women (LVM/ht^2.7 ^with a partition value of 51 g/ht^2.7 ^in both men and women). All the other criteria gave higher prevalence of abnormal LV geometry in women than in men. This is similar to the observations by Wachtell et al[19]. The most plausible reason for this is that the men in this study generally had larger BSA and were taller than their female counterparts. The later are however heavier and have larger BMI as noted in the baseline characteristics of the subjects in this study and in our previous reports[9].

Two recent publications have compared the prognostic implications of different normalization for LVH. Report from the Strong Heart Study group showed that the presence of LVH identified by LV mass normalized for height to allometric powers is associated with higher incident cardiovascular events than is LVH detected by normalization for body surface area[5]. In the second study conducted in a population of hypertensive subjects with low prevalence of obesity, population risk attributable to LV hypertrophy was similar for height as well as body surface area based partition values [4]. Large scale prospective study similar to these will surely be required in defining the prognostic implications of different LV geometry in native Africans.

### Limitations

The subjects used for this single centre cross-sectional study were unselected in terms of whether on treatment or not. Similar study will therefore be necessary in newly diagnosed untreated hypertensive Nigerians.

## Conclusion

LVH by echocardiography is present in 30.9–56.0% of hypertensive Nigerian seen in tertiary health care setting. Abnormal LV geometry is found in 61.1–74.0% of the subjects. Eccentric LV geometry is the commonest abnormal LV geometry in our subjects. Abnormal LV geometry is commoner in women and unevenly distributed in men and women except when the partition value of 51 g/ht^2.7 ^was used for both gender.

Partition values are strongly population dependent since they are derived from reference normal subjects. Different methods of indexation lead to different prevalence of LVH because the LV mass is looked at from different point of view. For example indexation to BSA offsets the independent impact of obesity on LV mass while indexation for height (especially when indexed to height raised to the power of 2.7) is useful in the definition of "genetically determined" LV mass.

Large scale prospective study will be needed to determine the prognostic implications of the different LV geometry in native Africans. We also intend to define which method of indexation is more reliable in our population through future prospective studies.

## Competing interests

The author(s) declare that they have no competing interests.

## Authors' contributions

AAA, OSO and AA conceived of the study and participated in the study design. OSO drafted the manuscript, AAA and OSO carried out the statistical analysis. OOO took part in the study design and study conception. DBO and AKA participated in the study design and data acquisition. AOF conceived of the study and participated in the study design. All authors read and approved the final manuscript.

## Pre-publication history

The pre-publication history for this paper can be accessed here:



## Figures and Tables

**Table 1:** Baseline clinical and demographic characteristics

**Parameter**	**All Subjects** **N = 457**	**Men** **N = 235(51.4%)**	**Women** **N = 222(48.6%)**	**P-value**
Age (yrs)	55.8 ± 13.3	56.0 ± 13.0	55.5 ± 13.5	0.672
Weight (kg)	73.1 ± 13.7	71.4 ± 13.5	74.8 ± 13.7	0.009
Height (cm)	164.3 ± 8.3	169.1 ± 6.9	159.3 ± 6.5	<0.0001
Body Mass Index (kg/m^2^)	27.1 ± 4.8	26.1 ± 4.4	28.1 ± 5.0	<0.0001
Body Surface Area (m^2^)	1.79 ± 0.18	1.85 ± 0.17	1.74 ± 0.16	<0.0001
Heart Rate (beats/min)	80.4 ± 16.7	80.2 ± 17.3	80.6 ± 16.1	0.774
Systolic Blood Pressure (mmHg)	142.7 ± 20.8	142.8 ± 21.0	142.7 ± 20.7	0.986
Diastolic Blood Pressure (mmHg)	88.3 ± 12.9	88.1 ± 12.8	88.5 ± 13.0	0.714
Pulse Pressure (mmHg)	54.3 ± 16.8	54.7 ± 17.8	54.0 ± 15.7	0.648
Mean Arterial Pressure (mmHg)	106.2 ± 14.8	106.3 ± 13.6	106.1 ± 15.9	0.887
%Overweight	27.8%	19.1%	36.9%	<0.0001

**Table 2:** Echocardiographic parameters

**Parameter**	**All Subjects** **N = 457**	**Men** **N = 235**	**Women** **N = 222**	**P-value**
Aorta (cm)	2.90 ± (0.43)	3.06 ± 0.42	2.74 ± 0.37	<0.0001
Left Atrium (cm)	3.51 ± (0.65)	3.59 ± 0.67	3.42 ± 0.62	0.004
IVSTd (cm)	1.04 ± (0.18)	1.08 ± 0.17	1.00 ± 0.18	<0.0001
PWTd (cm)	1.00 ± (0.19)	1.01 ± (0.17)	0.98 ± 0.20	0.55
LVEDD (cm)	4.82 ± 0.91	5.08 ± 0.96	4.54 ± 0.75	0.0001
LVESD (cm)	3.26 ± (0.95)	3.49 ± 1.04	3.01 ± 0.77	0.001
FS (%)	33.5 ± 8.1	32.7 ± 8.07	34.4 ± 8.13	0.032
EF (%)	69.4 ± 10.7	68.4 ± 10.9	70.3 ± 10.4	0.059

IVSTd= interventricular Septal Thickness in Diastole, PWTd= Posterior Wall Thickness in Diastole, LVEDD= Left Ventricular End-Diastolic Diameter, LVESD= Left Ventricular End-Systolic Diameter, FS= Fractional Shortening, EF= Ejection Fraction

**Table 3:** LV geometric patterns according to partition value

PARTITION VALUE	LVH/NO LVH	%LVM	NORMAL GEOMETRY (i)	CONCENTRIC REMODELLING (ii)	ECCENTRIC HYPERTROPHY (iii)	CONCENTRIC HYPERTROPHY (iv)	%ABNORMAL LV- GEOMETRY (ii + iii + iv)
LVM/BSA M/F = 116/104	221/236	48.4	133(29.1%)	103(22.5%)	125(27.4%)	96(21.0%)	70.9
LVM/BSA M/F = 125/125	141/316	50.3	178(38.9%)	138(30.2%)	80(17.5%)	61(13.4%)	73.5
LVM/BSA M/F = 125/110	180/277	56	159(34.9%)	118(25.8%)	99(21.7%)	81(17.6%)	74
LVM/BSA M/F = 131/110	208/249	30.9	138(30.2%)	111(24.3%)	120(26.3%)	88(19.1%)	61.1
LVM/HT M/F = 143/102	230/227	39.4	121(26.5%)	106(23.2%)	137(30.0%)	93(20.3%)	65.2
LVM/HT M/F = 126/105	242/215	45.5	120(26.2%)	95(20.8%)	138(30.2%)	104(22.8%)	69.8
LVM/HT^2.7^ M/F = 49.2/46.7	256/201	53	119(26.1%)	82(17.9%)	139(30.4%)	117(25.6%)	73.7
LVM/HT^2.7^ M/F = 51/51	211/246	46.2	140(30.6%)	106(23.2%)	118(25.8%)	93(20.4%)	69.4

LV = Left Ventricular, LVM = Increased Left Ventricular Mass, BSA = Body Surface Area, HT = Height, M/F = Male/Female.

**Table 4:** Distribution of LV geometry according to gender

Partition value	Normal geometry M/F	Concentric remodeling M/F	Eccentric Hypertrophy M/F	Concentric hypertrophy M/F	P-value
LVM/BSA M/F = 116/104	84/49	73/30	34/91	31/65	<0.0001
LVM/BSA M/F = 125/125	94/84	83/55	24/56	21/40	<0.0001
LVM/BSA M/F = 125/110	94/65	83/35	24/75	21/60	<0.0001
LVM/BSA M/F = 131/110	95/43	85/26	23/97	19/69	<0.0001
LVM/HT M/F = 143/102	90/31	87/19	28/109	17/76	<0.0001
LVM/HT M/F = 126/105	85/35	73/22	33/105	31/73	<0.0001
LVM/HT^2.7^ M/F = 49.2/46.7	64/55	54/28	54/85	50/67	0.0011
LVM/HT^2.7^ M/F = 51/51	69/71	58/48	49/69	46/47	0.261

LV = Left Ventricular, LVM = Left Ventricular Mass, BSA = Body Surface Area, HT = Height, M/F = Male/Female
